# TiC-carbide derived carbon electrolyte adsorption study by ways of X-ray scattering analysis

**DOI:** 10.1007/s40243-015-0059-4

**Published:** 2015-08-30

**Authors:** Lorie Trognko, Pierre Lecante, Nicolas Ratel-Ramond, Patrick Rozier, Barbara Daffos, Pierre-Louis Taberna, Patrice Simon

**Affiliations:** CIRIMAT, UMR-CNRS 5085, Université Toulouse III, Paul Sabatier, 31062 Toulouse Cedex 9, France; Réseau sur le Stockage Electrochimique de l’Energie (RS2E), FR CNRS 3459, Paris, France; CEMES, UPR-CNRS 8011, Université Toulouse III, Paul Sabatier, 31055 Toulouse Cedex 4, France

**Keywords:** Ion adsorption, Supercapacitors, Porous carbon, Ionic liquids, X-ray scattering

## Abstract

Understanding ion adsorption in nanoporous carbon electrodes is of great importance for designing the next-generation of high energy density electrical double-layer capacitors. In this work, X-ray scattering is used for investigating the impregnation of nanoporous carbons with electrolytes in the absence of applied potential. We are able to show that interactions between the carbon surface and electrolytes allow adsorption to take place in sub-nanopores, thus confirming experimentally for the first time the results predicted by molecular dynamic simulations.

## Introduction

Electrical double-layer capacitors (EDLCs), also known as supercapacitors, are one of the most promising electrochemical energy storage devices for high power delivery or energy harvesting applications [1]. While batteries, that use redox reactions to store charges in the bulk of active materials, are suitable for applications requiring energy lasting for several hours to days, EDLCs are power devices allowing the fast restitution of the energy stored. EDLCs store charges through the reversible adsorption of ions from an electrolyte onto high surface area carbons, thus charging the double-layer capacitance. Large-size electrochemical capacitors (ECs) are already used today for power supply and energy harvesting in various applications such as aeronautics, tramways, HEVs, etc [1]. The absence of faradic reactions confers to EDLCs high power density (up to 10 kW kg^−1^), medium energy density (5 Wh kg^−1^) and outstanding performances in cyclability (>1,000,000) [2]. One of the main challenges EDLCs are facing is to increase their energy density, given Eq. (1):

1 $$E = 1/ 2CV^{ 2} ,$$

where *E* is the energy (J), *C* the capacitance (F) and *V* the voltage (V). Such energy improvement can be achieved either by increasing their capacitance, playing with carbon properties [3–5] or by enlarging the electrochemical voltage window of the electrolyte [6–8].

Using carbide derived carbons (CDCs) with controlled, fine-tuned, narrow pore size distribution in the microporous range [9, 10], it was demonstrated that the maximum capacitance was achieved when the ion size was close to the carbon pore size [9, 11, 12]. Such result, defying the conventional wisdom that pores twice larger as the ion size were needed for optimizing the charge storage, was attributed to a shortening of the ion–wall pore distance [9].

Important efforts were directed towards the understanding of the ion adsorption and transport mechanisms in such confined pores. The capacitance increase was attributed to the absence of the over-screening effect [13, 14] and to a large electronic screening by the pore wall of like–like ions repulsive interactions [15]. Additionally, both the ion desolvation and the local charge stored at the carbon electrode increase in highly confined pores [16]. Still using modelling, it was also proposed that the electrolyte could wet—and access—small pores (<1 nm) even though no potential was applied at the electrode, suggesting that ion adsorption in these small pores was not an electrosorption-driven process [14]. However, to our knowledge apart adsorption in activated carbon of neutral molecule such as water [17], there is no experimental evidence of such spontaneous adsorption of charged species without applied potential.

X-ray scattering is a powerful technique for investigating both atomic ordering and electronic density contrast evolution linked with pore filling. Usually, the range of distances to be analysed is so different that, for technical reasons, two different experimental set up must be used: small angle X-ray scattering (SAXS) and wide angle X-ray scattering (WAXS). SAXS brings information about phenomena impacting large distance range such as pore filling, while WAXS is used to study short (interatomic) distances. However, the high capacitance observed in porous carbons being associated with the presence of micropores with size close to interatomic distances (between 0.6 and 1 nm), these sizes are small enough to generate “typical SAXS” patterns in the lowest part of the angular range normally used to investigate interatomic distances. That makes the use of the WAXS technique sufficient to investigate both pore filling and pore wall modifications. Moreover, the access to low and high angles parts in a single experiment allows an easy evaluation of the ratio between the different components (carbon, electrolyte, sample holder) useful to separate their contributions.

The scope of this paper is then to report an original use of the X-ray scattering technique to investigate the spontaneous adsorption of ions in pores of the same size. A preliminary study confirming that the use of WAXS technique allows determining both atomic ordering and pore filling using a single experiment is first presented. Then, the effectiveness of selected CDC impregnation with different electrolytes is studied giving for the first time experimental evidences of spontaneous adsorption of ions without applied potential.

### Experimental methods

Microporous carbon, purchased from Y-Carbon Company, was prepared by chlorination of titanium carbide as described elsewhere [9, 18]. The control of the annealing temperature allows selecting pore size of 0.66 and 0.9 nm as confirmed by BET technique [10]. The electrolytes (propylene carbonate from Aldrich, TEABF_4_ from Acros organics and RTIL from Solvionic) are used as received and stored under argon atmosphere in glovebox with <0.1 ppm content of water and O_2_. Samples for the X-ray scattering experiments were prepared by filling Lindemann glass capillaries of 1.5 mm diameter with carbon and electrolyte. Capillaries were centrifuged to ensure carbon being appropriately wet by the electrolyte.

Small-angle X-ray scattering data were recorded using an INEL SAXS device in transmission geometry with point focus using a sealed source at Cu Kα wavelength (*λ* = 0.154056 nm) and an image plate detector. The X-ray beam passes through Kirkpatrick–Baez mirrors, ensuring low divergence and spot-like shaping of the X-ray beam. Beam-size is adjusted using three consecutive cross-slits systems. The sample to detector distance was calibrated using a silver behenate reference sample. The collected intensity was corrected from capillary contribution and radial averaging was performed using the Fit2D software [19].

Wide angle X-ray scattering data were recorded in Debye–Scherrer geometry using a sealed source at Mo Kα wavelength (*λ* = 0.071069 nm) and a solid-state detector. This combination makes instrument background and absorption corrections close to negligible in our case. Data were thus only corrected for polarisation and capillary contribution.

## Results and discussion

### Validation of the “SWAXS” technique

**Fig. 1:**
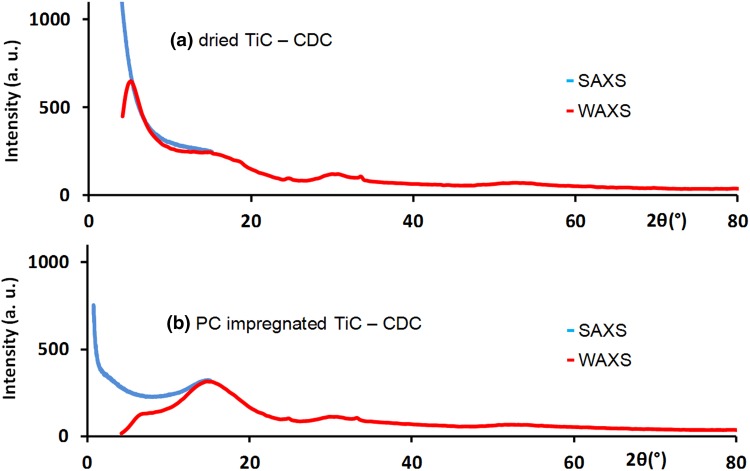
SAXS (*blue*) and WAXS (*red*) experimental intensity vs scattering angle for **a** dried and **b** PC impregnated TiC-CDC

CDC with 0.7 nm pore size and propylene carbonate was selected for this preliminary study. Both dried and PC impregnated CDC samples have been characterised by conventional SAXS and WAXS techniques. The corrected intensities are reported in Fig. 1. The comparison indicates that in both cases the low-angle part of WAXS data corresponds to the SAXS signal. Intensity of dried CDC was also simulated according to an excluded volume chain model [20] using Scatter software [21, 22] with the parameters summarized in Table 1.

**Table 1:** Parameters used in the excluded volume chain model

Radius of gyration	*v* (Flory exponent)	*γ*
0.375 nm	0.54	1.1

**Fig. 2:**
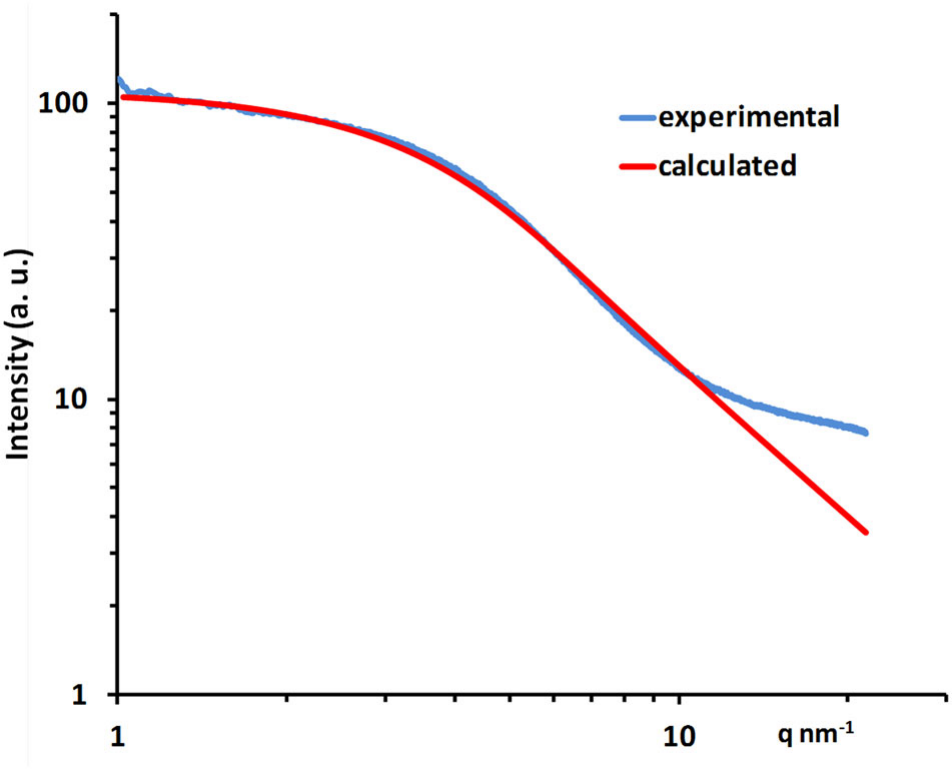
Experimental (*blue*) and calculated (*red*) SAXS intensity vs *q* (modulus of scattering vector) of dried TiC-CDC

Its comparison with SAXS experimental data (Fig. 2) shows good agreement and leads to a refined pore diameter of 0.75 nm (from the 0.375 nm radius of gyration and according to a spherical model) in good agreement with the value obtained by BET (0.66 nm). Deviation for large *q* values (above 10 nm^−1^) is related to WAXS contributions not taken in account in the model. The SAXS data of PC impregnated CDC show, compared to the dry CDC ones, a large decrease of scattered intensity at low angle indicating the decrease of electronic density contrast between pores and walls thus confirming the effectiveness of pore impregnation using PC. The low-angle part of the WAXS data exhibits the same intensity pattern, confirming, as expected from the pore size, that the typical SAXS signal extends up to angles large enough to be recorded using WAXS configuration. This confirms that both pore filling and atomic ordering evolutions of CDC with narrow pore size distribution below an average value of about 0.9 nm can be simultaneously studied using a single WAXS type experiment.

### Study of the CDC spontaneous impregnation

Once the approach was validated, the impregnation of CDC using different electrolytes selected for their respective behaviour was carried out. TiC CDC with pore size of 0.66 and 0.90 nm were used since these materials are known to show high capacitance [19]. To check their behaviour without any electric field imposed, different electrolytes such as 1-ethyl-3-methylimidazolium tetrafluoroborate (EMI, BF_4_) and 1 M tetraethylammonium tetrafluoroborate (TEA, BF_4_) in propylene carbonate (PC) are selected to offer a wide range of ion size in combination to the presence or absence of solvent.

The X-ray scattering spectra of the different samples (electrolytes, dry carbon and impregnated carbons) are collected and corrected as detailed in the experimental section. In all scattering spectra presented in the following figures, as expected from the study described above, the typical SAXS signal can be observed in the lowest scattering angle area of the measured WAXS data. The low-angle part (below 7°) will then be used as a conventional SAXS signal to follow the pore filling, while larger scattering angle domain (above 7°) will be used to follow atomic ordering evolution of CDC and ions during impregnation. As a general comment, apart the intensity of the signal directly related to the amount of CDC, there is no evolution of the peaks characteristics of the structure of the CDC. This clearly indicates that whatever the electrolyte used, and despite small ion-C distances, no significant change of the CDC walls’ constitution can be detected during the impregnation.

**Fig. 3:**
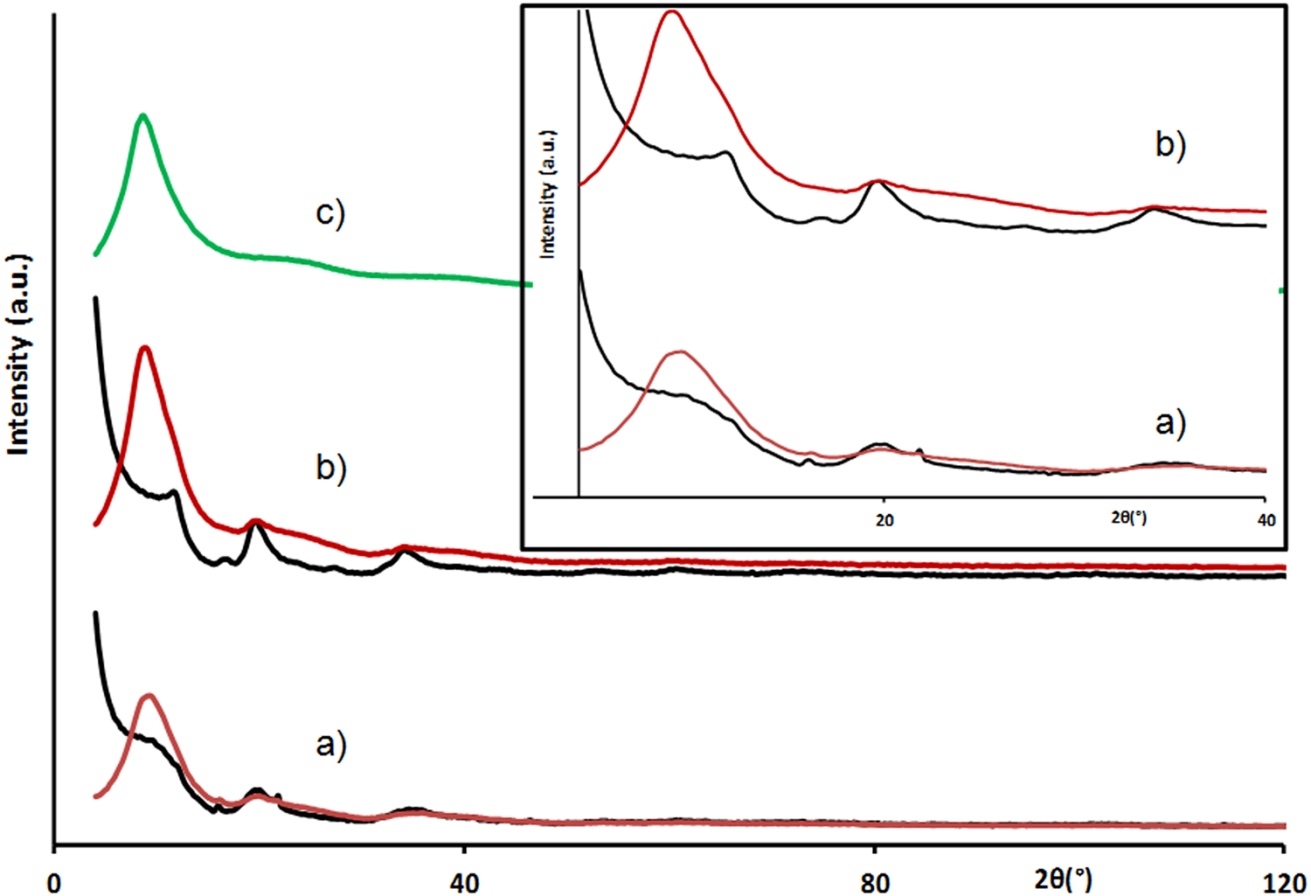
X-ray scattering spectra of impregnated carbon (*red*) and dry carbon (*black*) **a** 0.66 nm pore size carbon, **b** 0.90 nm pore size carbon and **c** 1 M TEA, BF4 in PC (*green*)

Because of its chemical stability at ambient temperature, the electrolyte 1 M TEA, BF_4_ in PC was selected and added to the two types of CDC. The X-ray scattering spectra recorded for the different impregnated samples are reported in Fig. 3 where the contribution from carbon alone (black), electrolyte alone (green) and carbon impregnated by the electrolyte (red) are presented.

**Fig. 4:**
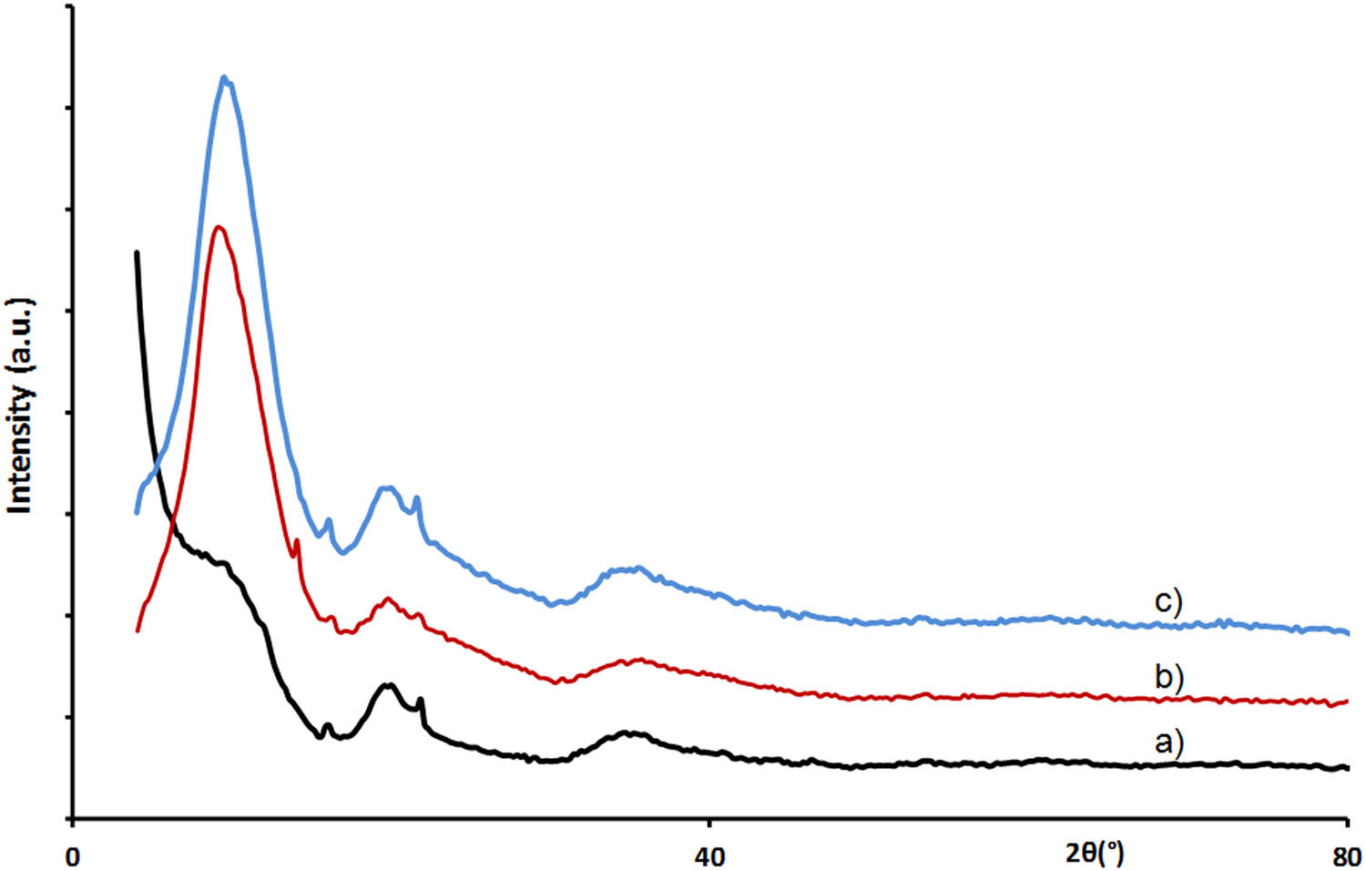
X-ray scattering spectra of 0.66 nm pore size carbon **a** dry, **b** impregnated by PC and **c** after impregnation and evaporation

The impregnation of carbon by 1 M TEA, BF_4_ in PC leads in both cases to a strong attenuation of scattered intensity for 2*θ* angles below 7°, related to the sub-nanometer pores’ characteristic length signature. This evolution evidences a decrease of the electronic density contrast between pores and their walls and indicates the adsorption of species in the sub-nanometer pore independently of steric limitations imposed by the pore size variation in agreement with reported results [23]. This clearly indicates the spontaneous adsorption of species even at null potential and constitutes the first experimental evidence confirming the results suggested by simulation [16]. However, because of the complexity of the studied electrolyte (solvent plus salt) it is necessary to determine whether salt, solvent or solvated salts are able to access the porosity (especially for 0.66 nm pore size). In order to discriminate the adsorption of salt from the solvent, a 0.66-nm pore size carbon is impregnated by PC only. The measured scattering signal (Fig. 4b) exhibits a decrease of the intensity in the low-angle part compared to the scattered signal of the dry CDC (Fig. 4a). It indicates the spontaneous filling of the pores by the solvent that can be associated with capillarity phenomenon in agreement with reported results on the adsorption of water in activated carbon [17]. In order to evidence the reversibility of this adsorption the sample is dried at 120 °C under static vacuum for 48 h. The data recorded for this impregnated then evaporated sample show (Fig. 4c) for large angular domain well-defined carbon structural peaks, thus confirming non adsorbed PC evaporation efficiency. Surprisingly, even if the carbon powder was dry in appearance, intensity drop at low angle is still present (Fig. 4c) indicating that sub-nanopores still contain PC.

These results indicate the existence of an interaction (whatever physical or chemical bonding) between carbon and PC molecules at the origin of the extra energy which would be necessary to remove adsorbed PC molecules. Unfortunately, this spontaneous pore loading with neutral and small molecules prevents studying of ion adsorption separately from the solvent contribution. To resolve this ambiguity, solvent-free electrolytes such as Room Temperature Ionic Liquids (RTIL) have been investigated. EMI, BF_4_ was selected thanks to its favourable X-ray signature, showing only small contributions at small scattering angles. Ion size of BF_4_^−^ is 0.48 nm and EMI^+^ is an anisotropic ion 0.76 nm long and 0.43 nm wide.

**Fig. 5:**
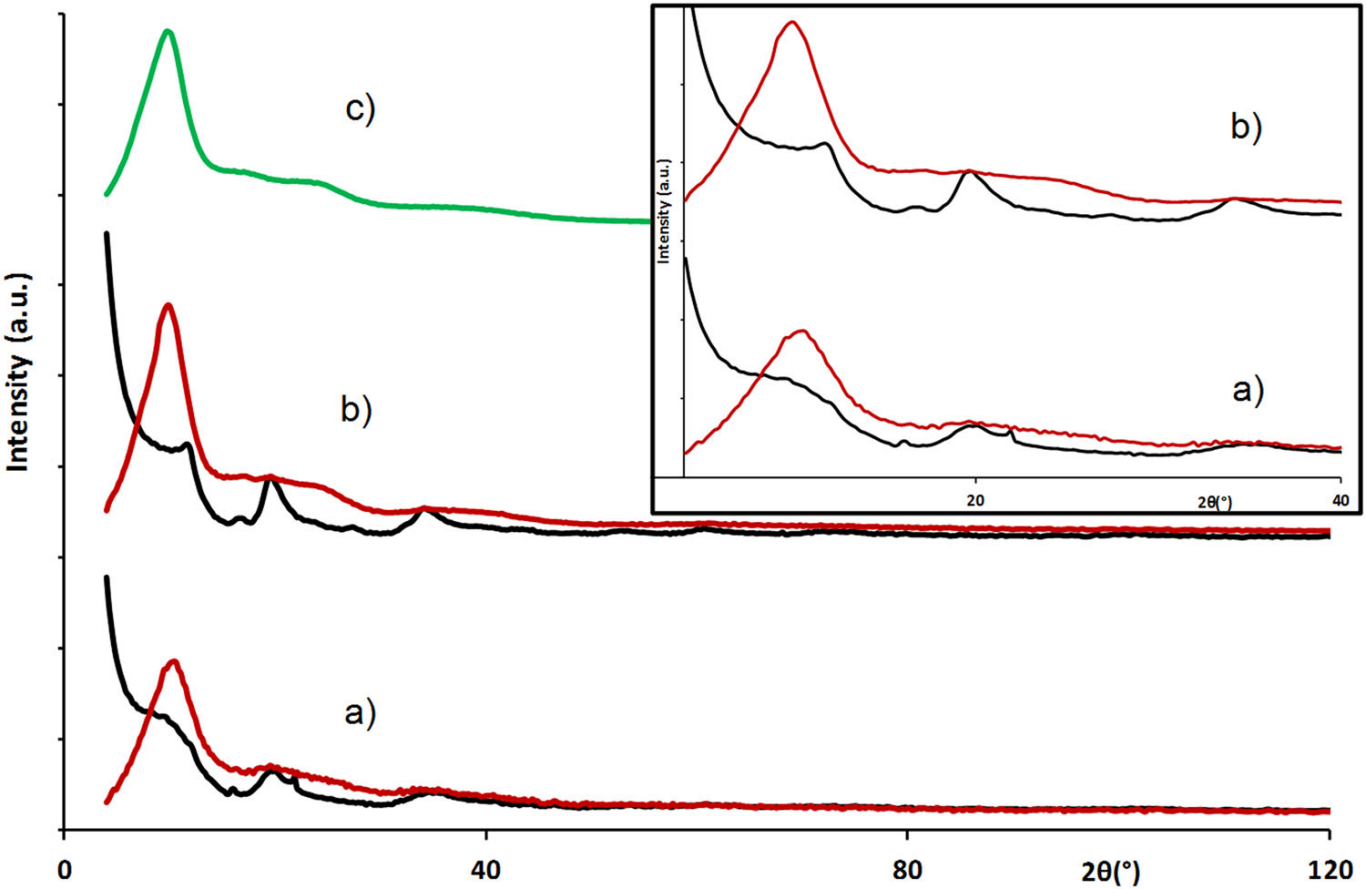
X-ray scattering spectra of dry carbons (*black*) and EMI, BF4 impregnated carbons (*red*) for **a** 0.66 nm pore size, **b** 0.90 nm pore size samples and of **c** EMI, BF4 ionic liquid (*green*)

On both X-ray scattering spectra (Fig. 5), intensity for angles <7° decreases, indicating pore impregnation by EMI, BF4 whatever the pore size. These results provide experimental evidence of the hypothesis deduced from molecular dynamic modelling [24] and more precisely of CDC porous carbon/RTIL interface modelling [16, 25] indicating the quasi instantaneous loading of sub-nanometer pores by charged species.

The structural peaks of carbon observed in the high-angle part remain unchanged after impregnation. That shows that despite interaction between adsorbed ions and pore walls, the structural arrangement of carbon is not significantly modified or that such modification should concern only atoms located near the surface.

## Conclusion

The reported results show that the filling of pores with size close to the one of adsorbed ions induces the shortening of the distances of interest, allowing the use of both SAXS and WAXS techniques using a single SWAXS experimental set up. The study of the evolution of the SAXS part of this SWAXS signal gives for the first time experimental evidences of spontaneous (without applied potential) filling of CDC pores without steric limitation. The study of the WAXS part of the SWAXS signal shows no significant changes in the structural arrangement of carbon. This is in agreement with whatever a localized interaction near the pore surface or the absence of strong chemical bonding between adsorbed species and pore walls. Despite that, an interaction should exist as indicated by the difficulties encountered to remove adsorbed species. The next step currently in progress is to develop cells for in situ experiments to study the behaviour of ions while a polarisation is applied between electrodes.
